# Characterization of Reclaimed Asphalt Pavement Material Properties for Hot In-Place Recycling

**DOI:** 10.3390/ma18050970

**Published:** 2025-02-21

**Authors:** Fangting Qu, Zhiyu Yang, Zhengnan Liu

**Affiliations:** 1 National Engineering Research Center of Highway Maintenance Technology, Changsha University of Science & Technology, Changsha 410114, China; 2 Hunan Communications Research Institute Co., Ltd., Changsha 410015, China

**Keywords:** hot in-place recycling (HIR), aging gradient, secondary aging, gradation failure, reclaimed asphalt pavement (RAP)

## Abstract

Asphalt with different aging degrees requires different rejuvenation methods. However, current applications of hot in-place recycling (HIR) for Reclaimed Asphalt Pavement (RAP) do not consider the differences in the aging degree of asphalt binder across different layers of RAP. Additionally, there is limited understanding of the changes in asphalt binder and aggregate properties during the HIR process. Changes in the properties of RAP materials can lead to inaccuracies in the mix design, potentially causing suboptimal performance. This study compares the performance of asphalt binders at different depths within RAP and clarifies the effects of the grinding and heating processes during HIR on both asphalt and aggregate properties. The aging gradient of RAP asphalt was assessed using macroscopic performance tests (bending beam rheometer (BBR), dynamic shear rheometer (DSR)) and microscopic techniques (scanning electron microscopy (SEM) and Fourier transform infrared spectroscopy (FTIR)). The effects of heating and milling on RAP materials were evaluated using conventional performance tests (DSR, BBR) and sieving analyses. The results show that the RAP asphalt exhibits an aging gradient under natural conditions, with the upper surface asphalt aging more than the lower layers. The heating process during HIR accelerates the secondary aging of RAP asphalt, reducing penetration by 25.3%, increasing the softening point by 7.4%, and decreasing ductility by 36.4%. The milling process causes gradation failure of RAP, with a damage rate of 14.4% of the coarse aggregates. Therefore, it is recommended that when using HIR for maintenance of severely aged pavements, the upper layer of the RAP should be separately milled and treated. The HIR mix design should consider the impact of heating and milling on RAP materials.

## 1. Introduction

During service, asphalt pavements are subjected to loads, UV radiation, heat, water, and oxygen, causing the asphalt film to gradually thin and age. This leads to various pavement diseases that severely affect driving safety and comfort and significantly reduce pavement durability [1,2]. As a result, pavement construction has progressively transitioned from a focus on new infrastructure development to an emphasis on the maintenance, rehabilitation, and preservation of existing roadways [3]. RAP is a significant byproduct produced when asphalt pavements reach the end of their functional lifespan. [4,5]. Meanwhile, the annual production of RAP in China continues to increase significantly. It is estimated that China produces 220 million tons of RAP each year during road maintenance processes [6]. Rising asphalt prices and growing environmental concerns have turned the recycling and reuse of RAP into a major area of research [7,8,9,10]. Recycling and crushing RAP, then adding new asphalt or rejuvenators for reuse, can effectively reduce the road industry’s reliance on non-renewable resources (asphalt and aggregates), resulting in significant environmental and economic benefits [11,12]. Long-term monitoring and analysis of HIR test sections in Florida, USA, indicate that the service life of recycled pavements can range from 7 to 14 years. Compared to reconstruction, HIR can save about 50% of initial construction costs and 40% of full life cycle costs [13,14]. HIR, known for its high recycling rate of old materials, low carbon emissions, and minimal disruption to traffic, has emerged as the most favored and widely adopted method for pavement recycling [15,16,17]. Consequently, in recent years, this technology has rapidly developed and found wide application. To improve the comprehensive technical system of HIR, scholars both domestically and internationally have conducted the following studies:

Yang Liu [18] found that preheating conditions significantly influence the performance and workability of new pavements, and by controlling the preheating temperature above 120 °C, better workability of the recycled asphalt mixture can be achieved. To enhance heating efficiency, Yuetan Ma et al. [19] developed a new method called the “ignition furnace method” to quantify the recycled asphalt content obtained during the HIR process and achieved results similar to those obtained using the FTIR testing method for activated RAP content. Hairong et al. [20] employed multi-stage heating technology to heat asphalt pavements. By comparing the temperature fields produced in asphalt pavements by multi-stage and single-stage heating technologies, they found that the rate of temperature increase with multi-stage heating was over 50% higher than that of single-stage heating. Additionally, researchers have introduced conductive materials into asphalt mixtures, making it possible to heat these mixtures using microwave heating. This method can significantly reduce heating time and rapidly increase the temperature of the asphalt mixture, with heating temperatures typically reaching up to 15–20 cm [21]. Pan et al. [22] reported that well-constructed HIR restores overall performance, including both high- and low-temperature stability, and that the compaction of asphalt pavements shows the most significant increase within the first 3 years of maintenance. Lixuelian et al. [23], based on image processing technology, proposed a quantitative characterization of the uniformity coefficient for HIR asphalt mixtures and found that the heating temperature of RAP has the most significant impact on the uniformity coefficient. The most noticeable change in the uniformity coefficient occurred when the heating temperature increased from 110 °C to 120 °C. Yuetan Ma et al. [24] determined the effective amount of rejuvenated asphalt by preparing mixtures with different proportions of rejuvenated and original asphalt. The amount of rejuvenated asphalt affects binder quality and the effective asphalt content. Asphalt properties influence the strength and cohesion of the mixture, while the effective asphalt content determines the mixture’s crack resistance.

Scholars primarily focus on the impact of mixing conditions (temperature, time) and construction control factors on the quality of HIR. Prior to the formal implementation of in-place hot recycling technology, a mixture design must be conducted to determine the proportions of virgin aggregates, virgin asphalt, and rejuvenators that meet the pavement’s performance requirements. The entire mixture design for HIR is based on the properties of RAP materials, and any changes in these properties can lead to inaccuracies in the design, potentially resulting in suboptimal performance. Therefore, accurately understanding the properties of RAP materials is essential when designing the mixture for HIR. Currently, the performance evaluation of RAP materials for HIR typically involves extraction tests using cold-cut road cores or pavement slabs to obtain aged asphalt and aggregates, followed by performance testing of the extracted asphalt and aggregates. This method of evaluating RAP materials overlooks the aging gradient of RAP and changes in RAP material properties during heating and milling of HIR, resulting in inaccurate and unreliable HIR mix designs that affect construction quality. Therefore, considering the aging gradient of the original pavement asphalt is essential during the construction and HIR mixture design processes.

To elucidate the performance variations in RAP materials across different layers and the changes in asphalt binder and aggregate characteristics during the RAP processing steps (heating and milling), with the goal of optimizing the design of recycled asphalt mixtures and enhancing the utilization efficiency of recycled asphalt materials. This study extracts asphalt binder from RAP at different depths, and the aging gradient in RAP is examined through a series of tests (DSR, BBR, FTIR, SEM). Additionally, RAP obtained from core drilling (CD RAP) and from heated milling (HM RAP) are analyzed to evaluate the effects of heating and milling on RAP properties in HIR. Conventional performance tests (DSR, BBR) and sieving analyses are employed to assess the impact on RAP materials.

## 2. Novelty and Contribution

This study has presented a comprehensive characterization of Reclaimed Asphalt Pavement (RAP) materials, with a particular focus on the aging gradient and the hot in-place recycling (HIR) process. To this end, core sampling from actual roadways has enabled an exploration of the varying aging degrees of RAP materials across different depths. In addition, both macroscopic and microscopic tests, including dynamic shear (DSR), bending beam rheometer (BBR), Fourier transform infrared spectroscopy (FTIR), and scanning electron microscopy (SEM), have been utilized to systematically reveal the aging characteristics of the asphalt binder in RAP and the impact of the HIR process on binder properties. Furthermore, this study has made recommendations for the treatment of RAP materials in the HIR process, emphasizing the necessity of layered milling for severely aged surface layers of RAP to ensure the optimization of the overall mixture performance.

## 3. Materials and Sample Preparation

This study used RAP from the Qinglan Expressway in Shandong Province, China. The RAP consisted of SBS-modified asphalt mixtures with a service life of over 10 years. The pavement structure included a 4 cm layer of modified asphalt stone mastic asphalt (SMA13), a 6 cm layer of medium-grain modified asphalt concrete (AC-20C), and an 8 cm layer of coarse-grain asphalt concrete (AC-25C).

### 3.1. Obtaining Asphalt from Different Depths of RAP

**Figure 1:**
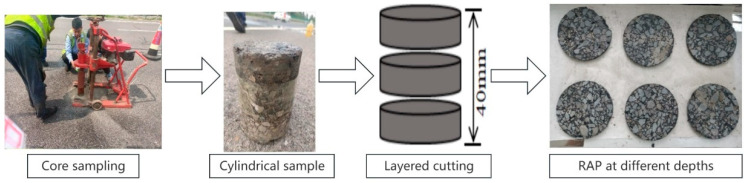
The process of obtaining RAP at different depths.

**Figure 2:**
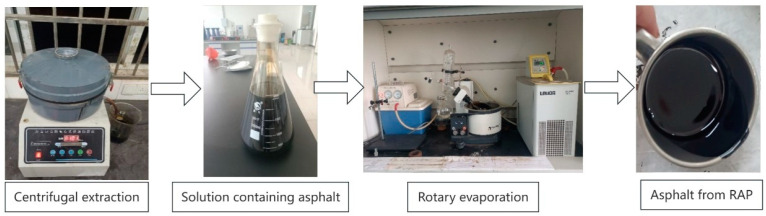
The process of extracting asphalt from RAP.

Wang et al. [25] observed that asphalt aging intensified over time, and pavement thickness significantly influenced the aging extent. Therefore, proper planning of the thickness for each layer was essential. Asphalt aging primarily results from thermal-oxidative aging, photoaging, and water-induced aging. UV radiation facilitated the aging degree of SBS-modified asphalt, with the depth directly impacted by UV radiation being approximately 0.1 mm [26,27]. Although UV radiation predominantly affected the surface of the asphalt, the aging depth could extend to 10 mm due to the diffusion of asphalt molecules and the presence of voids in the pavement layers [28]. Consequently, the thickness of each RAP mixture layer in this study was set to 10 mm. The procedure for extracting asphalt from different depths of RAP was as follows:

(1) Obtaining RAP at different depths.: The pavement underwent a 40 mm surface layer HIR treatment, so the depth range of the asphalt pavement aging gradient was 0–40 mm. The sampling method selected for RAP was core drilling, as shown in Figure 1. To ensure the samples contained 0–40 mm of RAP, the core drilling depth exceeded 40 mm.
(2) Preparation of core samples: The obtained core samples were initially cut, removing the portion below 40 mm, to obtain 0–40 mm depth core samples. The 0–40 mm core samples were then sectioned. The 40 mm core samples were cut into 10 mm thick sections, resulting in three 10 mm thick asphalt mixture sheets. The three sheets, from top to bottom, represented the asphalt mixture from the pavement surface layer at 0–10 mm, 15–25 mm, and 30–40 mm (designated as the upper, middle, and lower layer RAP, respectively). The cut sheets were cleaned of surface dirt and dried at room temperature.
(3) Asphalt extraction at different depths of RAP: The RAP mixture was placed in an oven at 80 °C for 15 min. After heating, the mixture sheets were separated. The asphalt extraction process is illustrated in Figure 2. Initially, trichloroethylene was utilized as the extraction agent, and the centrifugation extraction method was employed to extract asphalt from RAP, resulting in a solution containing asphalt, according to the ASTM D2172 [29]. Subsequently, the binder was then separated from TCE using a rotary evaporator, following the procedure outlined in ASTM D5404 [30], which yielded asphalt extracted from various depths of RAP.

### 3.2. Obtaining RAP Through Heated Milling and Core Sampling

**Figure 3:**
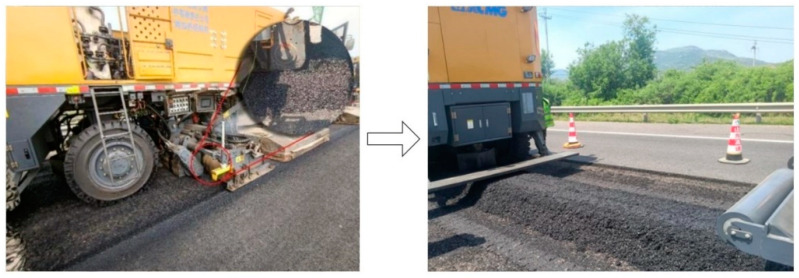
RAP is obtained through heating and milling.

To study the changes in the performance of RAP materials before and after HIR processing, it was necessary to use RAP materials that had been heated milled and those that had not been heated and milled in HIR. The methods for obtaining these two types of RAP materials are as follows:

(1) RAP obtained from heating and milling (HM RAP): The heated and milled RAP in this study was the surface layer of the road, 4 cm of SBS-modified asphalt mastic stone. The selected RAP was heated and softened by five hot air heaters and milled by one rotary milling machine, model XCMG JHM450 (Xuzhou, China). The specific milling parameters are presented in Table 1. After hot air heating, the road surface temperature reached 175 °C. The RAP mixture milled was gathered into piles by the milling machine’s gathering device, as shown in Figure 3. Therefore, the method for obtaining heated milled RAP involved directly shoveling RAP from the gathered piles.
(2) RAP obtained from core drilling (CD RAP): RAP was obtained using the core sampling method illustrated in Section 3.1. To ensure consistency between the depth of the RAP obtained by traditional methods and the above HM RAP, the core samples were transferred to an oven at 100 °C for 1 h. Once the time elapsed, the RAP mixture beneath 4 cm in depth was removed. To ensure the accuracy of the RAP gradation, the aggregate on the sides of the cylindrical core samples, which were damaged by core sampling, was stripped. After stripping, the remaining part was manually broken up, and the resulting mixture was RAP obtained from core drilling.
(3) Obtain asphalt and aggregates from two types of RAP: The RAP mixtures obtained from the two aforementioned methods were subjected to asphalt extraction according to ASTM D2172 [29]. This resulted in the extracted asphalt and aggregates from the HM RAP and CD RAP.

**Table 1:** Milling parameters.

Parameter	Type of Milling Surface	Milling Machine Travel Speed	Milling Depth	Rotor Speed
Values	SMA-13	2 m/min	40 mm	100 r/min

## 4. Tests

First, the aging gradient of the RAP was characterized by conducting macroscopic performance tests (DSR, BBR) and microscopic performance tests (FTIR, SEM) on asphalt at different depths of the RAP. Second, the impact of heating and milling in HIR on the performance of the RAP materials was studied through conventional performance tests (three major indicators of asphalt, DSR, BBR) and sieving tests.

### 4.1. Basic Properties Tests of Asphalt

The aged asphalt extracted from RAP was tested for softening point, ductility, and penetration according to ASTM (D5 D36 D113) [31,32,33]. Due to the brittleness of the extracted asphalt at 5 °C, the ductility test temperature was set to 15 °C with a loading rate of 5 cm/min.

### 4.2. Dynamic Shear Rheometer (DSR) Test

**Figure 4:**
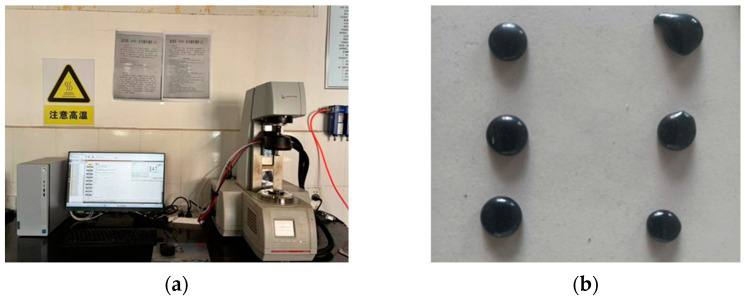
DSR test: (**a**) DSR; (**b**) scanning specimens.

A DSR model HR-10, shown in Figure 4a, was used for temperature scanning tests according to ASTM D7175 [34]. The test temperature range was 46–82 °C, the test fixture diameter was 8 mm, the angular frequency was 10 rad/s, and the strain level was set to 1%. Asphalt was prepared into 8 mm temperature scanning specimens, as shown in Figure 4b, and subjected to temperature scanning tests to measure the rutting factor (G*/sinδ) of the extracted asphalt.

### 4.3. Bending Beam Rheometer (BBR) Test

**Figure 5:**
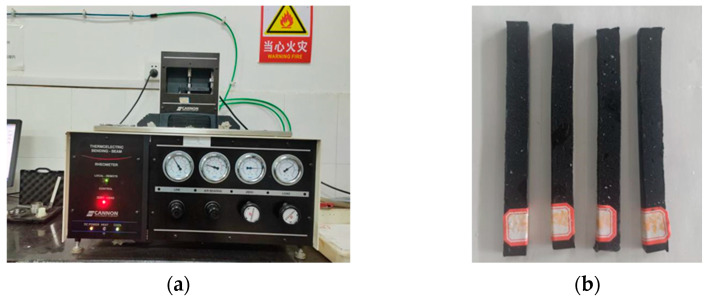
BBR test: (**a**) BBR; (**b**) Beam-shaped specimens.

The BBR model used in this study was the TE-BBR, as shown in Figure 5a. Asphalt was prepared into beam specimens (127 mm × 6.35 mm × 12.7 mm), as illustrated in Figure 5b. In accordance with ASTM D6648 [35], BBR tests were conducted on the beam specimens to measure the stiffness modulus (S) and creep rate (m) for the extracted asphalt, as per AASHTO T313. The calculation methods for S and m are provided in Equations (1) and (2).

(1) $$S\left(t\right)=\frac{PL^{3}}{4bh^{3}\delta (t)}$$

(2) $$m=\frac{d\left[logS(t)\right]}{d\left[log(t)\right]}$$

where S(t) represents the time-dependent flexural creep stiffness, P is the constant load, L is the span length, b is the width of the beam, h is the thickness of the beam, and δ(t) is the deflection of the beam.

### 4.4. Brookfield Viscosity Test

A Brookfield viscometer model NDJ-1F was employed to measure the viscosity variations in extracted asphalt from different depths of RAP, following ASTM D4402 [36]. Asphalt samples from different layers, each weighing 10 g, were placed into viscosity sample tubes. The samples were then tested on the viscometer to measure asphalt viscosity at temperatures of 135 °C and 170 °C.

### 4.5. Fourier Transform Infrared (FTIR) Test

This study used infrared spectroscopy to analyze the characteristic functional group differences in asphalt from each layer. The differences in characteristic functional groups reflect the aging degree differences in the extracted asphalt from each layer of the RAP. The infrared spectrometer used was a Thermo Scientific Nicolet Is 50 FTIR (Waltham, MA, USA). The extracted asphalt from different layers was placed in the infrared spectrometer for scanning analysis. A total of 32 scans were performed on each sample, with the scanning range set between 4000 and 500 cm^−1^.

### 4.6. Scanning Electron Microscope (SEM) Test

**Figure 6:**
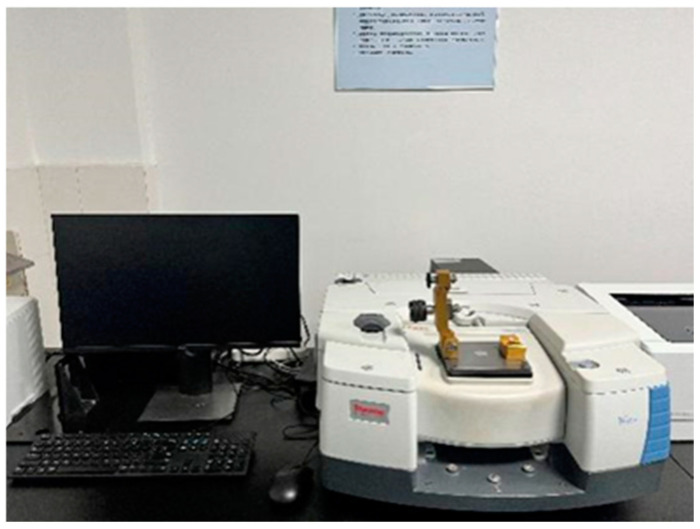
SEM.

The SEM model used was S-3000N, as presented in Figure 6. SEM tests are suitable for observing and analyzing surface morphology. The extracted asphalt from each layer was cut into 5 mm pieces with relatively flat surfaces. The asphalt pieces were gold-sputtered and then placed in the SEM for scanning.

### 4.7. Sieving Test

The loose HM RAP and CD RAP were subjected to centrifugal separation tests according to ASTM D2172 [29], resulting in the aggregate from RAP. The separated aggregate was subsequently dried at 105 °C for over 4 h, after which it was sieved following ASTM C136 [37].

## 5. Analysis of Tests

### 5.1. Analysis of Macroscopic Performance of Asphalt from Different Depths of the RAP

DSR, BBR, and viscosity tests were performed on the extracted asphalt from various depths. These three macroscopic performance tests assessed the shear deformation resistance at high temperatures, rheological properties at low temperatures, and the viscosity of the asphalt. By comparing the results of these tests, the degree of aging of the extracted asphalt can be determined.

#### 5.1.1. High-Temperature Performance of RAP Asphalt

**Figure 7:**
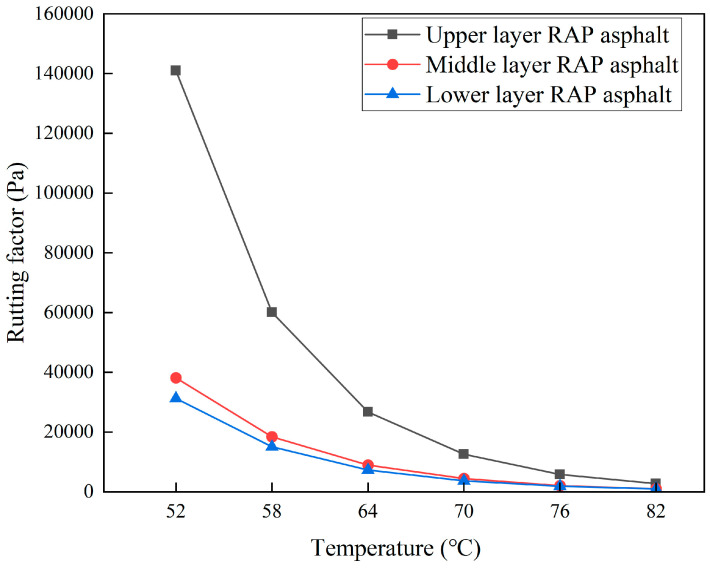
Rutting factors of RAP asphalt in different layers.

DSR tests were performed on asphalt extracted from different depths of the RAP, with the rutting factors of the RAP asphalt shown in Figure 7. The rutting factor of the RAP asphalt consistently decreases with increasing temperature, indicating a reduction in the asphalt’s resistance to deformation as the temperature rises. The rutting factor of the asphalt extracted from the upper layer of the RAP is substantially higher than that from the middle and lower layers, with the middle layer exhibiting a slightly higher value than the lower layer. This suggests that the high-temperature shear resistance of the asphalt in the upper layer significantly exceeds that of the asphalt in the middle and lower layers, with the middle layer’s resistance slightly greater than that of the lower layer.

The significantly higher rutting factor in the upper layer is attributed to its direct exposure to air, water, and ultraviolet rays, as well as its bearing of vehicle loads, all of which accelerate the aging process of the asphalt, thereby enhancing its high-temperature shear resistance. The slightly higher rutting factor of the extracted asphalt from the middle layer, as compared to the lower layer, can be explained by the RAP’s structure type, SMA-13, which has low porosity and high asphalt content, limiting the penetration of aging agents such as oxygen, water, and ultraviolet rays. The middle and lower layers of RAP are located in an anaerobic, waterless, and lightless environment, which nearly prevents direct aging. However, because the middle layer is adjacent to the upper layer, the significant aging difference between these layers facilitates the diffusion of aged asphalt molecules from the upper to the middle layer. The diffusion of aged asphalt molecules to the lower layer is minimal due to the comparable aging degree between the middle and lower layers, resulting in a slightly higher aging degree of the extracted asphalt from the middle layer compared to the lower layer.

#### 5.1.2. Low-Temperature Performance of RAP Asphalt

**Figure 8:**
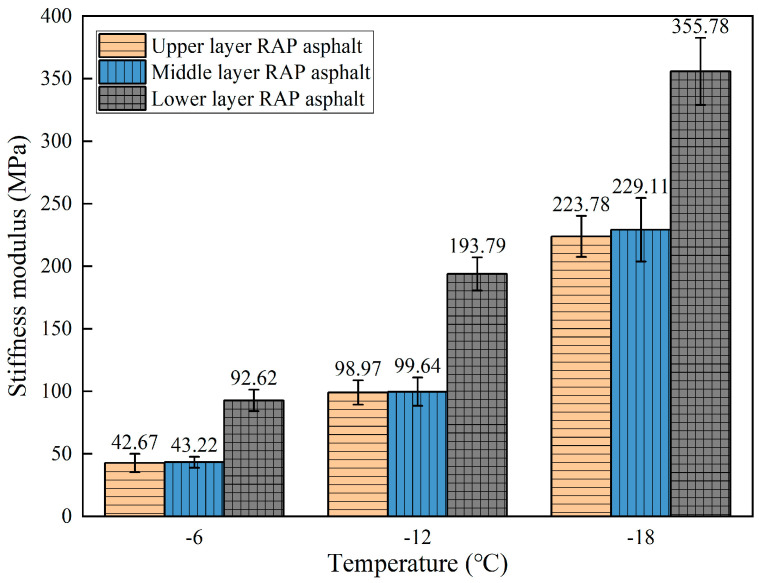
Stiffness modulus of RAP asphalt in different layers.

**Figure 9:**
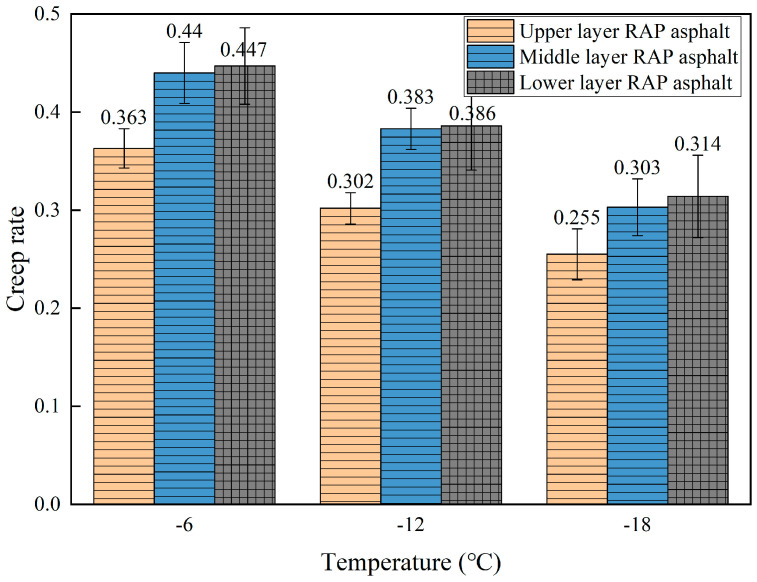
Creep rate of RAP asphalt in different layers.

BBR tests were performed on the beam specimens to measure the stiffness modulus (S) and creep rate (m) at 60 s for asphalt extracted from different depths of the RAP, as shown in Figure 8 and Figure 9. As the temperature decreases, the stiffness modulus (S) of the RAP asphalt gradually increases while the creep rate (m) decreases. This indicates that as the temperature drops, the asphalt becomes stiffer, and its stress relaxation ability diminishes, making it more prone to cracking. The stiffness modulus of the asphalt extracted from the upper layer of RAP is significantly higher at all three temperatures compared to that of the middle and lower layers, while the stiffness modulus of the asphalt extracted from the middle layer is comparable to that of the lower layer. In terms of creep rate, the asphalt from the upper layer exhibits the highest rate, followed by the middle layer, while the lower layer shows the lowest creep rate, with the middle and lower layers being comparable. This indicates that the low-temperature performance of the asphalt from the upper layer is the poorest, followed by the middle layer, which is slightly worse than the lower layer. The above results can be attributed to the fact that the upper layer mixture is directly exposed to aging factors such as air, sunlight, high temperatures, and loads, resulting in a higher degree of aging.

The low-temperature properties of asphalt decline with aging, which is why the asphalt extracted from the upper layer shows the poorest performance at low temperatures. The middle layer RAP is in contact with the upper layer mixture, causing the aged asphalt molecules from the upper layer to gradually diffuse downwards. This results in the extracted asphalt from the middle layer having a lower degree of aging than the upper layer but higher than the lower layer, and thus, its low-temperature performance is slightly worse than that of the lower layer.

#### 5.1.3. Brookfield Viscosity

**Figure 10:**
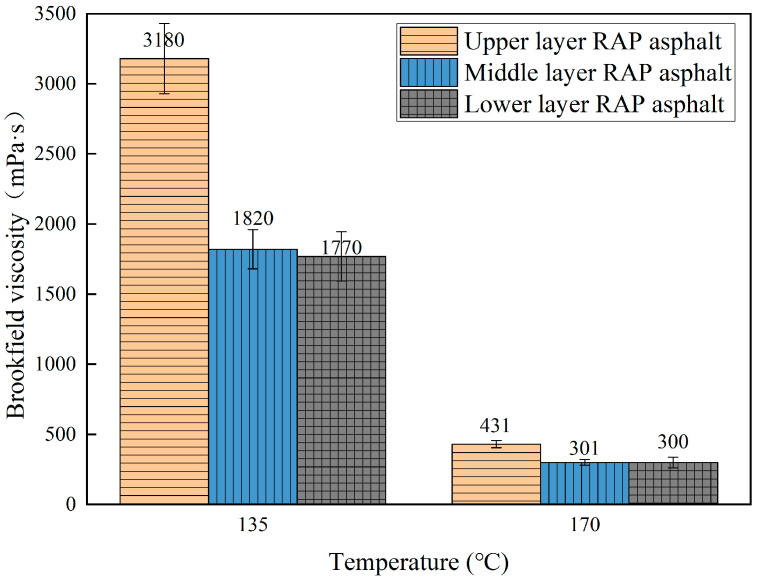
Brookfield viscosity of RAP asphalt in different layers.

Brookfield viscosity tests were conducted on the extracted asphalt from different depths of the RAP, with the viscosity values presented in Figure 10.

As the temperature increases, the viscosity of the extracted asphalt decreases due to the enhanced dissolution of lighter components, further influenced by aging. After long-term operation of the original pavement, the upper layer RAP directly exposed to aging sources has undergone significant aging. The middle layer RAP is affected by the diffusion of aged asphalt molecules from the upper layer, while the lower layer is almost unaffected by the environment and aged asphalt diffusion. This results in severe aging of the asphalt in the upper layer, with the middle layer asphalt aging slightly more than the lower layer asphalt.

### 5.2. Analysis of Microscopic Characteristics of Asphalt from Different Depths of the RAP

FTIR and SEM tests were conducted on the extracted asphalt from different RAP depths. These tests were used to verify the aging degree of RAP reflected by macroscopic performance from the perspective of asphalt functional groups and microscopic surface morphology.

#### 5.2.1. FTIR Test Results

**Figure 11:**
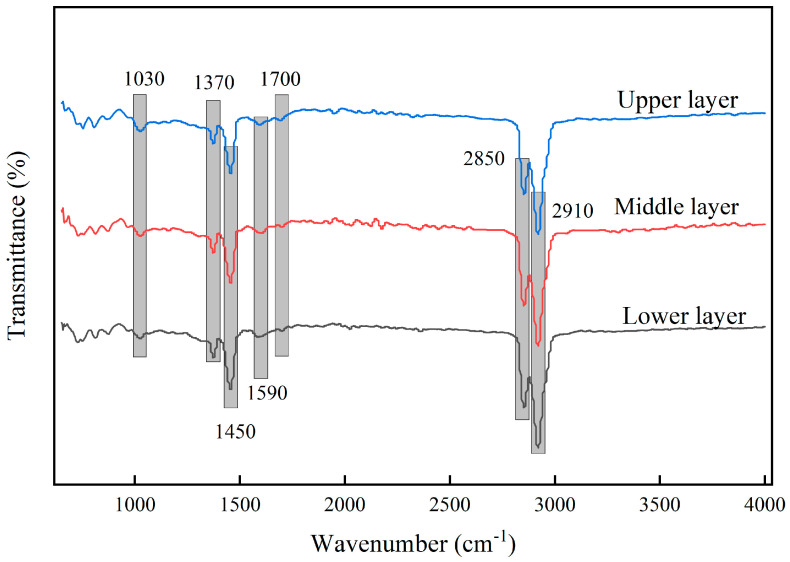
Infrared spectra of RAP asphalt in different layers.

FTIR has been established as an effective analytical method for detecting various functional groups in binders. FTIR tests were performed on asphalt extracted from different depths of the Reclaimed Asphalt Pavement (RAP). The infrared absorption spectra are shown in Figure 11, with the sulfoxide functional group index used as a quantitative indicator of asphalt aging and the carbonyl functional group index serving as a reference. [38]. The calculation methods are shown in Formulas (3) and (4). The peaks at 1370 cm^−1^ and 1590 cm^−1^ in the spectrum are attributed to aromatic ring skeletal vibrations. The peaks at 2850 cm^−1^ and 2910 cm^−1^ correspond to C-H stretching vibrations, which are caused by methyl (-CH_3_) and methylene (-CH_2_-) groups in the asphalt.

Sulfoxide functional group index:

(3) $$I_{S=O}=\frac{A_{1030{\mathrm{cm}}^{-1}}}{A_{1450{\mathrm{cm}}^{-1}}}$$

$I_{S=O}$ is the sulfoxide functional group index of the asphalt. $A_{1030{\mathrm{cm}}^{-1}}$ and $A_{1450{\mathrm{cm}}^{-1}}$ represent the peak areas of the absorption peaks at 1030 cm^−1^ and 1450 cm^−1^, respectively.

Carbonyl functional group index:

(4) $$I_{C=O}=\frac{A_{1700{\mathrm{cm}}^{-1}}}{A_{1450{\mathrm{cm}}^{-1}}}$$

$I_{C=O}$ is the carbonyl functional group index of the asphalt. $A_{1700{\mathrm{cm}}^{-1}}$ and $A_{1450{\mathrm{cm}}^{-1}}$ represent the peak areas of the absorption peaks at 1700 cm^−1^ and 1450 cm^−1^, respectively.

The peak areas of the saturated C-H (1450 cm^−1^), sulfoxide (1030 cm^−1^), and carbonyl (1700 cm^−1^) absorption peaks for asphalt extracted from different layers of the Reclaimed Asphalt Pavement (RAP) are presented in Table 2.

By substituting the corresponding absorption peak areas from Table 2 into Formulas (1) and (2), the sulfoxide and carbonyl indices for asphalt from different RAP layers were calculated, as shown in Table 3. The sulfoxide index of the asphalt extracted from the upper layer is 53.8% higher than that of the middle layer and 62.5% higher than that of the lower layer. The sulfoxide index of the asphalt extracted from the middle layer is 15.8% higher than that of the lower layer. The carbonyl index of the asphalt extracted from the upper layer is 31.3% higher than that of the middle layer and 46.5% higher than that of the lower layer, with the middle layer showing an 11.6% higher carbonyl index than the lower layer. These results indicate that the aging degree of the asphalt extracted from the upper layer is significantly greater than that of the middle and lower layers, while the middle layer shows a slightly higher aging degree than the lower layer. This finding is similar to the aging index variation calculated by Raqiqa et al. using laboratory-aged asphalt mixtures for 6 years, where the aging degree of the upper layer asphalt mixture was significantly higher than that of the middle and lower layers, and the aging degree of the middle layer was only slightly higher than that of the lower layer [39]. The FTIR results align with the macroscopic performance results.

#### 5.2.2. SEM Results Analysis

**Figure 12:**
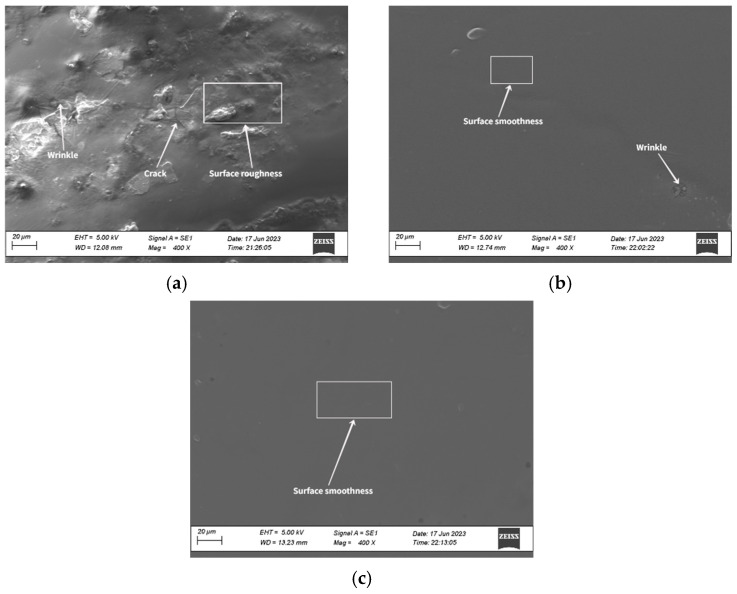
Surface morphology of RAP asphalt in different layers: (**a**) upper layer, (**b**) middle layer, (**c**) lower layer.

The SEM results of the extracted asphalt from different depths of the RAP are shown in Figure 12. Figure 12a shows that the surface of the asphalt binder extracted from the upper layer of RAP is rough, exhibiting wrinkles and cracks. The asphalt binder from the middle layer has a smoother surface, with only subtle wrinkling, while the binder from the lower layer of RAP displays a flat and smooth surface, resembling a more homogeneous material. The dissolution of SBS polymer particles in asphalt primarily relies on the absorption of the light components within the asphalt. When the light components are insufficient, the SBS polymer chains tend to curl, causing phase separation with the asphalt. Therefore, the blocky substances observed on the surface of the asphalt in the upper layer of RAP may be related to the morphological changes in SBS: the higher aging degree of the upper-layer asphalt results in a lower content of light components, which affects the swelling of SBS in the asphalt, leading to a decrease in compatibility between SBS and asphalt, causing molecular chain aggregation and the formation of raised structures on the surface. Additionally, as asphalt ages, its fluidity decreases and stiffness increases, making it more prone to brittle failure under external forces, resulting in the appearance of microcracks on the surface. For the asphalt binder in the middle and lower layers of RAP, the lower exposure to UV radiation and limited oxygen involved in the aging process result in a lower degree of aging. As shown in Figure 12b,c, the surfaces are smoother, with only minor wrinkling observed in the asphalt binder from the middle layer of RAP.

### 5.3. The Effect of Heating and Milling in HIR on the Asphalt Properties of RAP

To characterize the impact of heating and milling on the properties of aged asphalt from RAP, the extracted asphalt was subjected to the three major indicators test, dynamic shear rheology test, and BBR test.

#### 5.3.1. Three Major Indicators

The basic properties test results are shown in Table 4. As seen in Table 4, both types of extracted asphalt have lower penetration, higher softening points, and reduced ductility, indicating significant aging due to exposure to UV radiation, heat, water, oxygen, and loads over the years. Compared to the CD RAP asphalt, the HM RAP asphalt showed a 25.3% reduction in penetration, a 7.4% increase in softening point, and a 36.4% decrease in ductility. This is because, during the heating of the RAP in HIR, heat is gradually transferred from the top to the bottom. The surface temperature of the pavement often differs from the bottom temperature after hot air heating. To ensure proper milling, the bottom surface temperature needs to reach the required level. Continuous heating of the pavement to achieve this leads to severe secondary aging of the upper asphalt layer. Consequently, the upper asphalt layer undergoes significant secondary aging due to prolonged high-temperature heating, resulting in reduced penetration and ductility and an increased softening point.

#### 5.3.2. High-Temperature Performance

**Figure 13:**
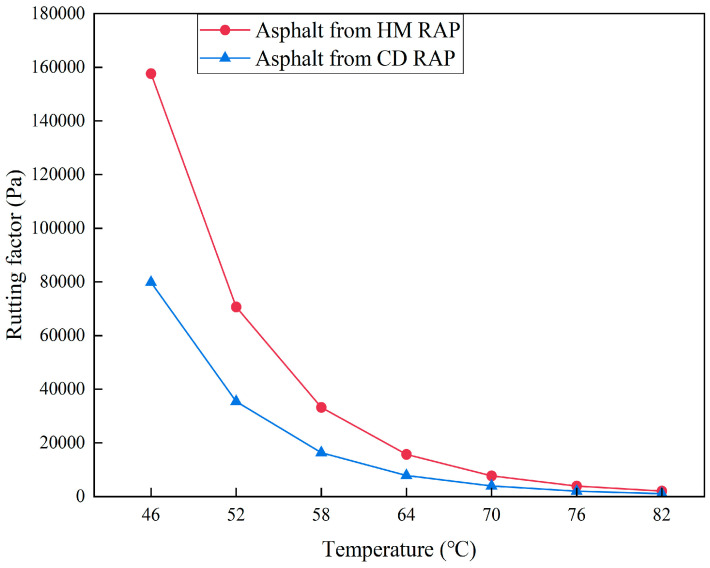
Rutting factors of asphalt from HM RAP and CD RAP.

DSR tests were performed on the extracted asphalt from both HM RAP and CD RAP. The rutting factors of the HM RAP asphalt and CD RAP asphalt are shown in Figure 13. The rutting factors of both types of RAP asphalt decrease gradually with increasing temperature. This is because higher temperatures reduce the shear deformation resistance of asphalt, making intermolecular movement more active. Thermal motion of molecules at high temperatures weakens intermolecular interactions, causing the asphalt to transition from a solid state to a viscous state with reduced deformation resistance, manifested as a gradual decrease in the rutting factor with rising temperature.

From 46 to 82 °C, the rutting factor of the HM RAP asphalt is greater than that of CD RAP asphalt, indicating that the HM RAP asphalt is harder and less prone to deformation at high temperatures. This is due to the RAP undergoing continuous heating before milling in HIR, leading to secondary aging of the upper layer RAP. This aging process transforms the composition of the asphalt from lighter to heavier fractions and its structure from sol–gel to gel-like, enhancing its elastic properties and making it harder. Therefore, the extracted asphalt from heated milled RAP has stronger shear deformation resistance at high temperatures, and its rutting factor increases with the degree of asphalt aging.

#### 5.3.3. Low-Temperature Performance

**Figure 14:**
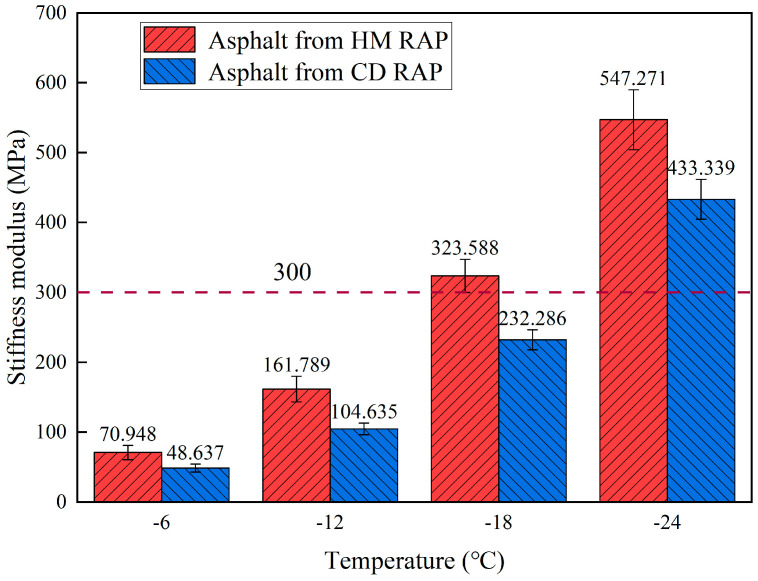
Stiffness modulus of asphalt from HM RAP and CD RAP.

**Figure 15:**
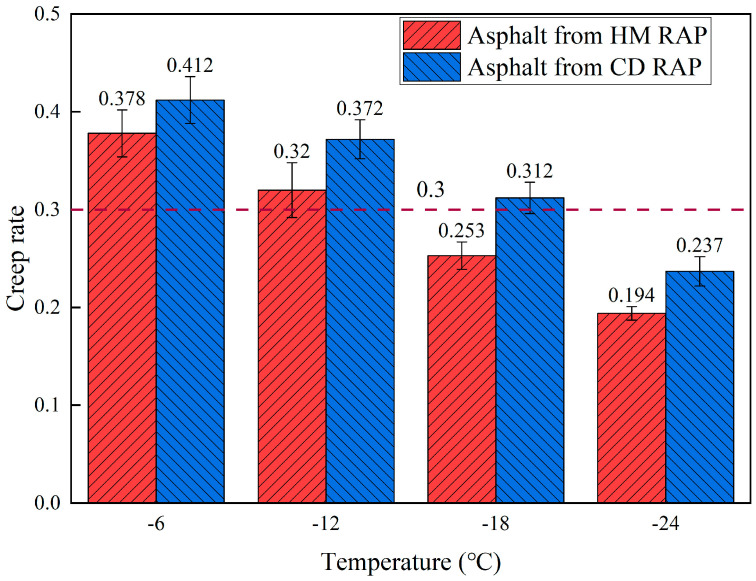
Creep rate of asphalt from HM RAP and CD RAP.

BBR tests were performed on the extracted asphalt from both CD RAP and HM RAP. The stiffness modulus (S) and creep rate (m) of the HM RAP asphalt and CD RAP asphalt are shown in Figure 14 and Figure 15, respectively. As shown in Figure 14 and Figure 15, as the temperature increases, the stiffness modulus of both types of RAP asphalt rises while the creep rate decreases. This is because lower temperatures reduce intermolecular movement within the asphalt, restricting molecular chain motion and enhancing intermolecular interactions, resulting in a more stable internal structure and decreased rheological properties, thus increasing the stiffness modulus and decreasing the creep rate. The stiffness modulus of HM RAP asphalt is greater than that of CD RAP extracted asphalt at all four temperatures, and its creep rate is lower than that of CD RAP.

The reason for these results is that the upper layer of CD RAP asphalt undergoes sustained high-temperature heating during the HIR heating process, leading to secondary aging. Aging of the asphalt causes the light components to transform into heavy components, increasing the proportion of asphaltenes in the asphalt material. This enhances the intermolecular interactions within the asphalt, resulting in a continuous extension of the stress relaxation time. As the degree of aging intensifies, the stress dissipation capability of the asphalt diminishes. This change reduces the plasticity and ductility of the asphalt while increasing its hardness and elasticity, leading to a rise in stiffness modulus and a decrease in creep rate under low-temperature conditions.

### 5.4. Impact of Heating Milling on the RAP Aggregates Gradation in HIR

To explore the effect of the heating milling process on the gradation of RAP aggregates, a sieving test was conducted on the extracted aggregates from CD RAP and HM RAP. The results of the sieving test were calculated using Formulas (5) and (6).

(5) $$a_{i}=\frac{m_{i}}{m}\times 100$$

$a_{i}$ is the percentage of retained aggregate for the i-th sieve size (%). $m_{i}$ is the mass of aggregate retained on the i-th sieve (g). *m* is the total mass of the sample.

(6) $$P_{i}=100-\sum_{m=i}^{16}a_{m}$$

$P_{i}$ is the passing percentage for the i-th sieve size (%). $a_{m}$ is the retained percentage for the m-th sieve size (%). *i* is the sieve size.

**Figure 16:**
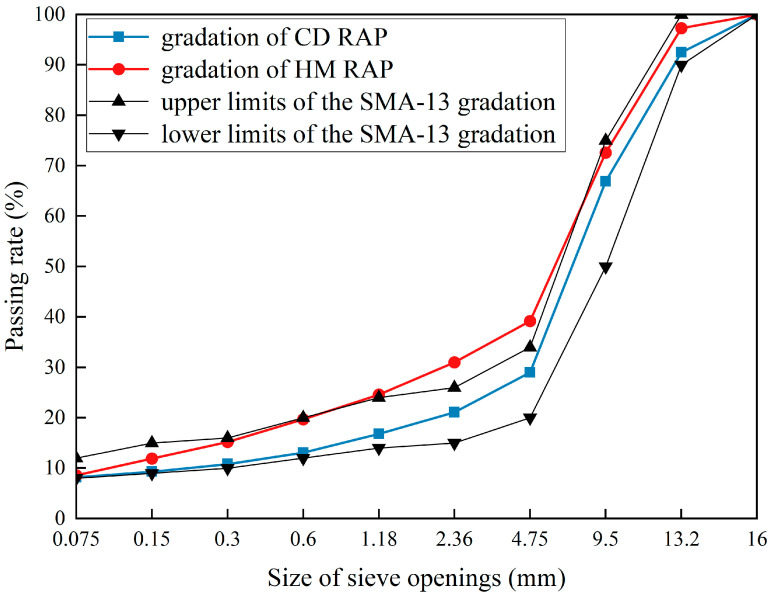
Gradation of HM RAP and CD RAP.

To analyze the gradation differences more intuitively between CD RAP and HM RAP, the gradation curves of the two RAP aggregates and the upper and lower limits of the SMA-13 gradation were plotted, as shown in Figure 16. The gradation curve of the CD RAP is within the upper and lower limits of the SMA-13 specification, whereas the gradation curve of the HM RAP is not. These results indicate that the milling process affects the RAP gradation, meeting the specification before milling but failing to meet it afterward.

The retained percentages for each sieve size ≥ 4.75 mm in the HM RAP are lower than those in the CD RAP, while the retained percentages for each sieve size < 4.75 mm in the HM RAP are higher than those in the CD RAP. This suggests that the milling process mainly damages the coarse aggregate with particle sizes ≥ 4.75 mm. The coarse aggregate is broken down into fine aggregate with particle sizes < 4.75 mm by the rotor milling, increasing the retained percentages of fine aggregate compared to the CD RAP. The cumulative retained percentage of coarse aggregate > 4.75 mm is 60.8% for HM RAP, while it is 71% for CD RAP, indicating that milling damages 14.4% of the total coarse aggregate. The severe damage to coarse aggregate during milling is due to the continuous heating of the pavement before milling. Since heat is gradually transferred from the top to the bottom, the upper part of the RAP heats up quickly, while the bottom heats up more slowly. This causes the upper RAP to reach the required milling temperature, sufficiently softening the asphalt, while the lower pavement does not reach the milling temperature, keeping the asphalt viscous. When the high-speed rotating rotor contacts the lower aggregate, the force from the rotor causes the aggregate to detach from the RAP. However, due to the high viscosity of the lower RAP asphalt, the aggregate does not break at the asphalt-aggregate bond but rather at the contact point with the rotor, where stress concentration causes the coarse aggregate itself to break.

## 6. Conclusions

Based on the performance test results of RAP materials obtained at different depths and using different methods, the following conclusions can be drawn:

(1) Through performance testing and micro-characteristic analysis of extracted asphalts from different layers of the RAP, it was revealed that the RAP asphalt exhibits an aging gradient under natural service conditions. Specifically, the degree of aging decreases from the surface downward, resulting in improved low-temperature performance, reduced high-temperature performance, and viscosity downward from the surface. Moreover, the aged asphalt is mainly concentrated in the upper part of the pavement surface layer, with the aging degree of the remaining parts being relatively mild.
(2) A series of performance tests on HM and CD RAP indicated that the heating process of HIR causes secondary aging of the asphalt, reducing the penetration of the extracted asphalt by 25.3%, increasing the softening point by 7.4%, and decreasing ductility by 36.4%. This enhances the high-temperature performance of the asphalt while weakening its low-temperature performance. Furthermore, the milling process of HIR damages coarse aggregates, where the amount of damaged coarse aggregate accounts for 14.4% of the total coarse aggregate.
(3) In HIR technology, the RAP exhibits an aging gradient, with the asphalt in the upper RAP being more severely aged. Heating of HIR significantly increases the degree of aging in the upper RAP asphalt. Therefore, it is recommended that HIR technology consider milling and processing the top 1 cm of the severely aged pavement (where extracted asphalt penetration is less than 20) separately to ensure optimal overall performance of the pavement.
(4) After heating and milling the RAP, the aging degree of the upper RAP asphalt significantly increases, and the coarse aggregate in the lower part is damaged, resulting in changes to the RAP gradation. Therefore, HIR mix design should not directly rely on the performance of core samples or cold-milled RAP. During construction, the aging degree of the pavement at different depths should be assessed first. After layered milling, RAP materials with different aging levels should be stored separately. The amount of new asphalt or rejuvenating agent added should be adjusted based on the degree of aging to optimize resource savings.

## 7. Limitations and Recommendations

This study focused on asphalt pavements with a single porosity value. Given the importance of porosity in the aging process, the effects of varying porosity on the aging gradient of asphalt pavements have not been explored. A more comprehensive analysis involving different porosity levels could provide a deeper understanding of how porosity influences the aging characteristics of asphalt binders. To better understand the relationship between porosity and aging in asphalt pavements, future studies should investigate asphalt pavements with varying porosity levels. Additionally, laboratory experiments should be conducted to analyze the diffusion of oxygen within asphalt mixtures.

## Figures and Tables

**Table 2:** Area of different absorption peaks of asphalt in each layer.

Layer of Mixture	Peak Area at 1030 cm^−1^	Peak Area at 1700 cm^−1^	Peak Area at 1450 cm^−1^
Upper Layer	166.399	40.140	640.656
Middle Layer	112.135	31.823	663.876
Lower Layer	102.700	30.592	705.712

**Table 3:** Functional group indices of asphalt in each layer.

Layer of Mixture	Sulfonyl Index $I_{S=O}$	Carbonyl Index $I_{C=O}$
Upper Layer	0.260	0.063
Middle Layer	0.169	0.048
Lower Layer	0.146	0.043

**Table 4:** Physical properties of extracted asphalt from RAP.

Physical Properties	HM RAP	CD RAP	Standard Code
Softening Point (TR&B)/°C	72.2	67.2	ASTM D36 [32]
Penetration (25 °C, 100 g, 5 s)/0.1 mm	21.5	28.8	ASTM D5 [31]
Ductility (15 °C, 5 cm/min)/cm	62.9	98.9	ASTM D113 [33]

## Data Availability

The original contributions presented in this study are included in the article. Further inquiries can be directed to the corresponding author.
